# Validity and Reliability of the Newly Developed Surface Electromyography Device for Measuring Muscle Activity during Voluntary Isometric Contraction

**DOI:** 10.1155/2018/4068493

**Published:** 2018-08-29

**Authors:** Myung Hun Jang, Se Jin Ahn, Jun Woo Lee, Min-Hyung Rhee, Dasom Chae, Jinmi Kim, Myung Jun Shin

**Affiliations:** ^1^Department of Rehabilitation Medicine, Pusan National University Hospital, Pusan National University School of Medicine, Busan, Republic of Korea; ^2^Division of Energy and Electric Engineering, Uiduk University, Gyeongju, Republic of Korea; ^3^School of Mechanical Engineering, Pusan National University, Busan, Republic of Korea; ^4^Department of Rehabilitation Medicine, Pusan National University Hospital, Busan, Republic of Korea; ^5^Biomedical Research Institute, Pusan National University Hospital, Busan, Republic of Korea; ^6^Department of Biostatistics, Clinical Trial Center, Biomedical Research Institute, Pusan National University Hospital, Busan, Republic of Korea

## Abstract

**Objective:**

The purpose of this study was to establish the validity and reliability of the newly developed surface electromyography (sEMG) device (PSL-EMG-Tr1) compared with a conventional sEMG device (BTS-FREEEMG1000).

**Methods:**

In total, 20 healthy participants (10 males, age 30.3 ± 2.9 years; 10 females, age 22.3 ± 2.7 years) were recruited. EMG signals were recorded simultaneously on two devices during three different isometric contractions (maximal voluntary isometric contraction (MVIC, 40% MVIC, 80% MVIC)). Two trials were performed, and the same session was repeated after 1 week. EMG amplitude recorded from the dominant biceps brachii (BB) and rectus femoris (RF) muscles was analyzed for reliability using intrasession intraclass correlation coefficient (ICC). Concurrent validity of the two devices was determined using Pearson's correlation coefficient.

**Results:**

Nonnormalized sEMG data showed moderate to very high reliability for all three contraction levels (ICC = 0.832–0.937 (BB); ICC = 0.814–0.957 (RF)). Normalized sEMG values showed no to high reliability (ICC = 0.030–0.831 (BB); ICC = 0.547–0.828 (RF)). sEMG signals recorded by the PSL-EMG-Tr1 showed good to excellent validity compared with the BTS-FREEEMG1000, at 40% MVIC (*r* = 0.943 (BB), *r* = 0.940 (RF)) and 80% MVIC (*r* = 0.983 (BB); *r* = 0.763 (RF)).

**Conclusions:**

The PSL-EMG-Tr1 was performed with acceptable validity. Furthermore, the high accessibility and portability of the device are useful in adjusting the type and intensity of exercise.

## 1. Introduction

Sarcopenia is defined as decreased skeletal muscle mass and muscle strength with age. Muscle mass and strength gradually decrease after reaching a peak in early adulthood, and the degree of decrease varies among individuals [1]. Elderly people with sarcopenia have a much higher fall risk and lower physical performance than do nonsarcopenic individuals [2]. Decreased muscle strength also reduces functional capacity and is a major cause of disability, mortality, and other adverse health outcomes [3, 4]. Because of individual differences, it is important to reduce the rate at which muscle mass declines to avoid premature sarcopenia. Sarcopenia can be evaluated by measuring skeletal muscle mass. It is common practice to examine the cross-sectional area, thickness, and weight of muscles using magnetic resonance imaging (MRI), computed tomography (CT), anthropometry, bioelectrical impedance analysis (BIA), and ultrasound. Muscle mass and strength are reduced in the third decade, and the prevalence of sarcopenia can be increased by the presence of obesity and the amount of physical activity. Therefore, managing the risk factors of sarcopenia through exercise is important in young and healthy adults [1, 5]. In addition, low physical performance can be assessed using functional measurements such as gait speed (e.g., 4 minute walking test) and grip strength [4, 6]. Muscle quality may be more important than muscle size in estimating the risk of falling, and monitoring muscle activity during daily activities can help in preventing sarcopenia and estimating the degree of frailty [7, 8]. It is also important to evaluate muscle quality in healthy elderly people before and after exercise and according to age [9, 10]. Muscle activity can be monitored and muscle quality can be evaluated, through surface electromyography (sEMG) [11, 12]. However, the sEMG devices developed so far are expensive and difficult to operate, which limits their use by nonspecialists. Therefore, a new sEMG device , that is, simple to use and highly accessible has been developed for people who are not familiar with EMG. The purpose of this study was to establish the validity and reliability of the new device.

## 2. Materials and Methods

### 2.1. Experimental Protocol

In total, 20 healthy participants (10 males, 10 females) between the ages of 21 and 34 years (males, age 30.3 ± 2.9 years, height 171.9 ± 3.8 cm, weight 74.1 ± 11.3 kg, body mass index (BMI) 25.4 ± 3.33 kg/m^2^; females, age 22.3 ± 2.7 years, height 162.1 ± 5.0 cm, weight 56.4 ± 5.0 kg, BMI 21.5 ± 1.9 kg/m^2^; mean ± SD) were recruited; all participants who provided informed consent prior to the study were recruited. Ethical approval was granted, and the informed consent form was approved by the Ethics Committee of Pusan National University Hospital, Busan, Korea (IRB number: 1703-018-052). Exclusion criteria included musculoskeletal disease, cardiopulmonary disease, and other diseases that could prevent exercise.

**Figure 1:**
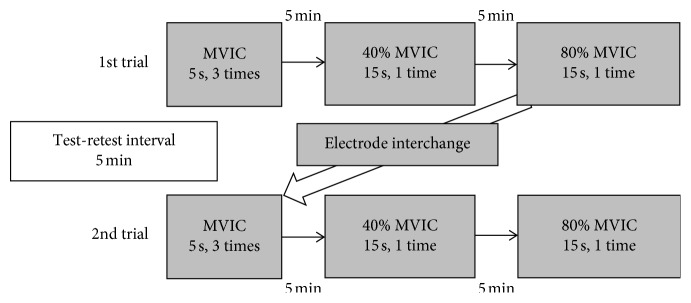
Scheme of the experimental protocol consisted of MVIC, 40%, and 80% MVIC. The same test was repeated after 1 week on the biceps brachii and rectus femoris muscles.

At each session, participants were first required to perform three maximal voluntary isometric contractions (MVIC) for 5 seconds each, with a 5-minute rest between contractions. Each session consisted of two trials. After three MVIC measurements, 15-second isometric contractions were performed at different intensity levels. In the pretest, it took at least 10–15 seconds to maintain the same intensity isometric contraction through visual feedback. First, 40% MVIC was performed, followed by 80% MVIC after 5 minutes of rest. In the second trial, the placement of the electrodes for the two devices (BTS-FREEEMG1000 and newly developed device) was interchanged, and contractions were again measured by the same method (Figure 1). During the test, participants received visual feedback about their performance from a monitor, which enabled them to maintain the muscle contraction at the target intensity. The same procedure was employed for the biceps brachii (BB) and rectus femoris (RF) muscles [13–15]. All tests were performed only with the dominant arm and leg. Participants were tested twice, with a week between sessions.

### 2.2. Mechanical Recording

The participants sat on a Biodex System 3 PRO dynamometer (Biodex Medical Systems, Shirley, NY, USA) with a visual torque feedback monitor. Each participant sat in an upright posture and was strapped firmly to the chair with adjustable belts across the arm, trunk, and thigh. To evaluate BB muscle contractions, the participant sat with the dominant arm flexed at 90° and the forearm flexed at 120° relative to the upper arm. To evaluate quadriceps (RF) contractions, the hips were flexed at 90° and the tested knee was flexed at 45°. The axis of the dynamometer was positioned at the center of the tested elbow or knee joint. The lever arm was fixed by the precalibrated force sensor [16].

### 2.3. EMG Recording

**Figure 2:**
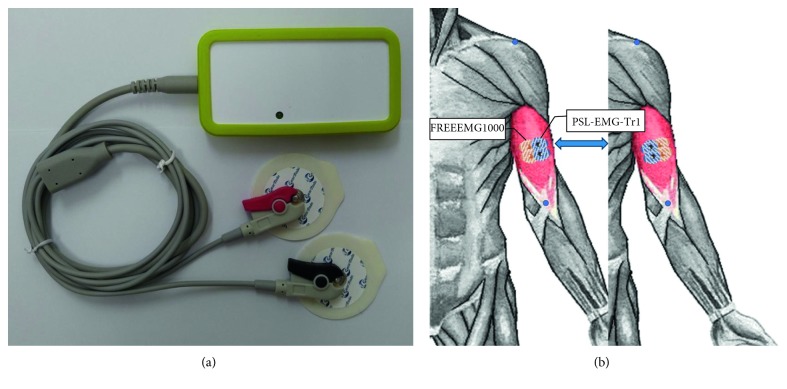
(a) The newly developed electromyography (EMG) machine (PSL-EMG-Tr1, PhysioLab Co., Ltd., Busan, Korea). (b) Placement of the two pairs of surface electrodes on the biceps brachii muscle.

The EMG signal was recorded simultaneously using two different sEMG devices. The BTS-FREEEMG (BTS-FREEEMG1000; BTS Bioengineering, Milan, Italy) was set to a sampling rate of 1,000 Hz per channel, and the signals were band-pass filtered from 20 to 500 Hz. The newly developed sEMG device (PSL-EMG-Tr1; PhysioLab Co., Ltd., Busan, Korea) was set to a sampling rate of 30,000 Hz, and signals were amplified with a 3–2,000 Hz bandwidth (Figure 2(a)).

Adhesive hydrogel surface electrodes (35 mm teardrop-shaped Kendall™ 200 Foam Electrodes; Medtronic, Minneapolis, MN, USA) were used, and the interelectrode distance, electrode placement procedure, and skin preparation followed standard Surface Electromyography for the Non-Invasive Assessment of Muscles (SENIAM) guidelines [17]. Two pairs of surface electrodes were attached parallel to the muscle fibers at an interelectrode distance of 2.0 cm. The distance between the pairs of electrodes was also 2.0 cm. After the first trial, the second trial was performed by interchanging the positions of the two pairs of electrodes of the each EMG devices (Figure 2(b)). After the interchange of the electrodes, the average of the values was used to compare the concurrent validity of the two devices.

The root mean square (RMS) value was used to analyze and process the recorded electrical signals in the muscles. Based on the square root calculation, the RMS reflects the mean power of the signal and is the preferred recommendation for smoothing. The RMS value can be used as a parameter to reflect the physiological activities of the motor unit during muscle contraction [18].

### 2.4. Statistical Analysis

Sample size was calculated using G^*∗*^Power software (ver. 3.1; Heinrich–Heine Universität, Düsseldorf, Germany). In this study, the number of subjects required for a null-correlation (R0) = 0, alternative correlation (R1) = 0.6, alpha = 5%, power = 80%, and two-tailed test value was 19. Statistical analysis was performed using SPSS software (ver. 18.0; SPSS Inc., Chicago, IL, USA). To determine concurrent validity between the two sEMG machines, Pearson's correlation coefficient (*r*) was used for the average of two trials in each device. Interpretation of the correlation coefficients was based on guidelines for Pearson's coefficients suggested by Portney and Watkins [19]: *r* > 0.75, good to excellent correlation; *r* = 0.50–0.75, moderate to good correlation; *r* = 0.25–0.50, fair correlation; and *r* = 0.00–0.25, little to no relationship [19]. The Bland–Altman plot was used to visually compare the mean values of the two trials in each device. Mean differences were calculated by subtracting the % MVIC of the PSL-EMG-Tr1 from the % MVIC of the BTS-FREEEMG1000. Limits of agreements (LOA) were calculated by using 2 standard deviations around the mean difference.

The intraclass correlation coefficient (ICC) of two trials performed on each week was used to indicate the relative reliability of the measurements. For the test-retest reliability, ICC using a two-way mixed-effects model and absolute agreement definition is used [20]. Munro's descriptors for reliability coefficients were used to index the degree of reliability: very high correlation, 0.90–1.00; high correlation, 0.70–0.89; moderate correlation, 0.50–0.69; low correlation, 0.26–0.49; and little or no correlation, 0.00–0.25 [21]. The paired *t*-test was also conducted comparing the RMS (*μ*V) and torque (N·m) between two trials of each week. Biodex is a device that has proved its reliability and validity. Therefore, Biodex was used only to evaluate the exact intensity during muscle contraction, and validity was compared between two sEMG devices [22].

## 3. Results

### 3.1. Torque Measurements

Twenty participants completed a total of four trials over 2 weeks.  Table 1 shows the peak torque (N·m) values at three isometric contractions levels (MVIC, 40% MVIC, and 80% MVIC) for the first and second weeks. Very high relative reliability was found at all three isometric contraction levels for both muscles (ICC: 0.985–0.994 for BB; 0.948–0.981 for RF).

### 3.2. Amplitude of sEMG

Recorded sEMG data were processed for RMS analysis. The amplitudes (*μ*V) of the nonnormalized RMS values recorded by the PSL-EMG-Tr1 devices at the three contractions levels for the first and second weeks are shown in Table 1. Moderate to very high relative reliability was found for all three contraction levels in both muscles (ICC: 0.832–0.937 for BB; 0.814–0.957 for RF). Overall, the reliability at various contraction levels was slightly lower for the PSL-EMG-Tr1 than for the Biodex device. This may be because the muscle group generating the torque includes other muscles in addition to the one measured by sEMG; this is discussed further below.

To compare EMG activity in the same muscle on different days or different individuals, or to compare EMG activity between muscles, the EMG must be normalized. Normalization of EMG signals (% MVIC) is shown in Table 2. Normalization of EMG signals is performed by dividing the EMG signals during the submaximal isometric contraction by a maximal EMG signal (MVIC). The normalized RMS values showed no to high relative reliability in BB and moderate to high relative reliability in RF (ICC: 0.030–0.831 for BB; 0.547–0.828 for RF). There were no statistical differences of normalized RMS values for two sEMG devices between the first and second trial (*p* > 0.05; Table 2).

### 3.3. Validity

**Figure 3:**
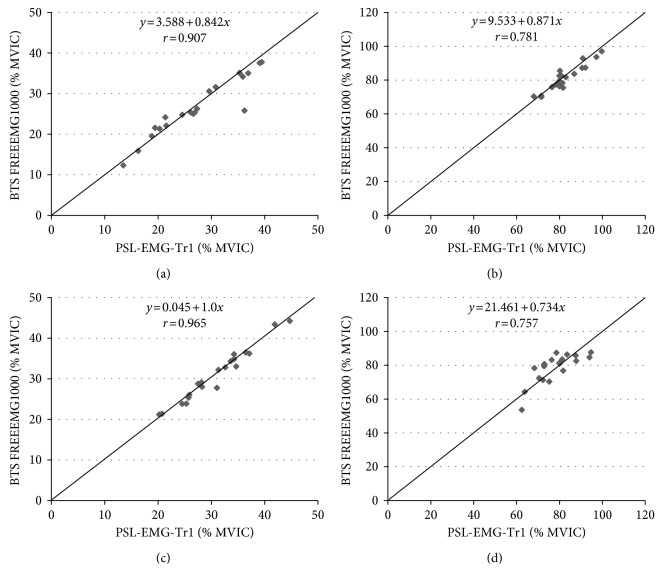
The relationship between BTS-FREEEMG1000 and PSL-EMG-Tr1 data. Data from 20 participants, for a total of 20 points in each plot. MVIC, maximum voluntary isometric contraction. (a) 40% MVIC (biceps brachii); (b) 80% MVIC (biceps brachii); (c) 40% MVIC (rectus femoris); (d) 80% MVIC (rectus femoris).

**Figure 4:**
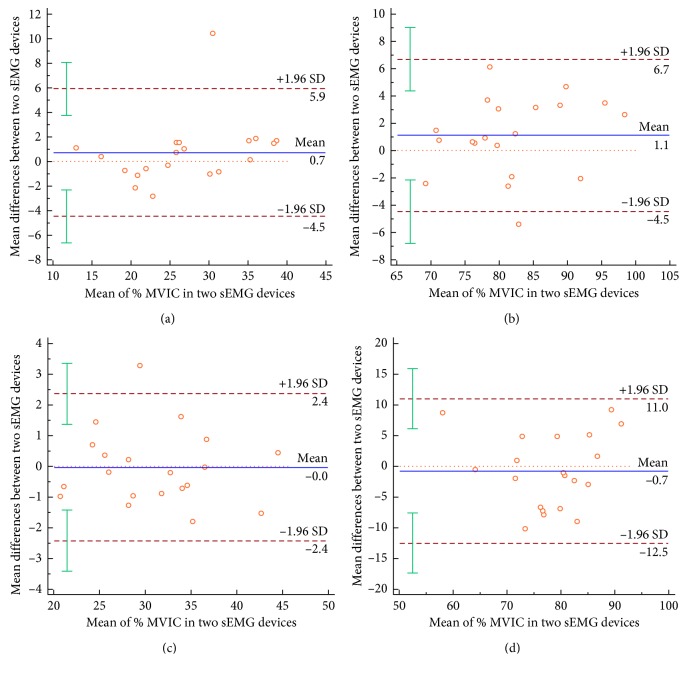
Agreement between BTS-FREEEMG1000 and PSL-EMG-Tr1. Means on the *x*-axis are the average of two sEMG devices for % MVIC; differences on the *y*-axis are the difference between the two devices. The 95% limits of agreement (LOA) are depicted (dashed lines). The error bars represent the 95% confidence interval for both the upper and lower limits of agreement. The 95% LOA include zero, indicating no systematic bias in performance between the two devices. (a) 40% MVIC (biceps brachii); (b) 80% MVIC (biceps brachii); (c) 40% MVIC (rectus femoris); (d) 80% MVIC (rectus femoris).

Figure 3 shows the validity of the two sEMG devices. Pearson's *r* values were used to evaluate validity because all of the % MVIC values measured in the BB and RF muscles were normally distributed. The two sEMG devices were compared using averages of all four trials for two weeks of 40% MVIC and 80% MVIC. The 40% MVIC displayed excellent validity for BB (*r* = 0.907) and RF (*r* = 0.965), and the 80% MVIC showed good to excellent validity for BB (*r* = 0.781) and RF (*r* = 0.757).  Figure 4 shows Bland–Altman plots which show the dispersion of the % MVIC of the two sEMG devices. The mean difference in % MVIC was small (0.0–1.1) with the majority of the data points within the 95% limits of agreement.

## 4. Discussion

In this study, we confirmed the reliability and validity of the newly developed sEMG device for monitoring muscle activity during exercise and in daily life. The nonnormalized RMS values measured on both devices showed high reliability (ICC: 0.832–0.937 for BB; 0.814–0.957 for RF). The normalized RMS values showed good to excellent validity (*r* = 0.781–0.907 for BB; *r* = 0.757–0.965 for RF) and showed nonsignificant results on the paired *t*-test (*p* > 0.05). Especially in the BB muscle, there was little reliability because of the low ICC of normalized RMS values.

Gaudet et al. reported the intersession reliability of maximal contraction of the elbow flexor group (BB, brachialis, and brachioradialis muscles) using sEMG. In this study, a relatively low ICC (range: 0.57–0.80) was seen in the BB in intersession single measurement. However, the average of repeated sEMG measurement was considered to obtain high reliability rather than single measurement [23]. Kollmitzer et al. evaluated the intersession reliability of measures of the knee extensor group (RF, vastus lateralis, and medialis muscles) and found that the overall reliability was good, especially for the RF muscle [16]. To improve reliability, testing in the lower limb can be a better choice than the upper limb and requires repeated measurements rather than a single measurement. Our study also confirmed higher ICC in RF than in BB. And our study compared the single measurements in each trial, which is one of the reasons for the low ICC. Based on previous studies and our results, it seems that EMG signal reproducibility has a great effect on the selection of certain muscles in upper and lower limbs.

In elderly populations, physical activity is reduced, with less than one-fifth of elderly individuals engaging in the recommended level of physical activity [24]. Commercially available computer-based physical activity monitors have been developed in recent years; these devices can enhance motivation in elderly individuals, record physical activity, and support physical exercise [25, 26]. In frail elderly persons, single-repetition maximum resistance training (RT) at 30% MVIC or greater can significantly improve muscle strength, muscle power, and functional outcomes. Therefore, supervised and controlled RT can be an effective measure against frailty [27]. RT also has neuromuscular benefits, and changes in muscle quality can be monitored via sEMG [28]. Applying wearable devise to monitor physical activities is good for feasibility and effectiveness and can encourage exercise through self-monitoring and goal setting [29]. Developing sEMG devices with high accessibility will therefore be very useful in establishing therapeutic strategies and evaluating the muscle condition of elderly people. The newly developed sEMG device is small in size with low cost, and therefore has good portability (Table 3). These advantages can also increase the accessibility of muscle monitoring during various physical activities or exercise.

To evaluate the accuracy of the new device, we recruited young people free of disease and disability. However, elderly individuals may have chronic diseases that could cause peripheral neuropathy or myopathy due to disuse atrophy, so the quality of surface EMG data may vary. However, such difficulties would not indicate a problem with the accuracy of the device itself. In other words, it is possible to compare muscle activity within one individual, but caution is needed when comparing individuals with one another. Despite these limitations, it is very encouraging that there is a tool that allows easy and objective evaluation of muscle quality in elderly people. For the clinical application of the device, further studies will be necessary for healthy elderly or sacropenic patients. This study had the other limitation that should be addressed: the electrodes were attached according to the SENIAM guidelines, but it is considered that the 2.0 cm interelectrode distance was the main cause of the lower ICC in this study. However, this problem will not affect the clinical application of muscle monitoring over time for people undergoing monitoring or engaging in exercise, as only one device will be used. This is because ICC was also lower in clinically widely used sEMG device (BTS-FREEEMG1000), and good to excellent validity was found in normalized RMS values of the two sEMG devices.

The newly developed sEMG device is wired for a single channel, although a wireless model for two or more channels is under development. A high sampling rate was used for the sEMG device in this study. Since most signals from the human muscles have frequency characteristics that are valid at less than 400 Hz, we recommend that the signal analysis sample more than twice the major quard of interest frequency. While we do not need this sampling frequency for existing RMS and MDF analysis, we are interested in signal characteristics that we have not known before by increasing the maximum sampling frequency. This will be used for further study of the characteristics of EMG signals of damaged muscles. Therefore, the newly developed instrument is measuring at a sampling frequency much higher than the frequency of interest. However, since many burdens are expected in the signal processing during commercialization, the sampling rate will be reduced to 2,000 Hz; it will enable fast signal processing and long recording time. In addition, if the EMG signals from the patient's muscles are automatically stored and the system is programmed so that this information can be delivered to medical staff located elsewhere, the therapeutic value of the device will increase still further.

## 5. Conclusion

Signals from the BB and RF muscles, recorded by the newly developed PSL-EMG-Tr1 device, showed good to excellent validity and moderate to high ICC values with nonnormalized RMS values. However, low ICC values were seen with the normalized RMS values. Although the sEMG itself may have limitations, it can be overcome somewhat through repeated measurements and appropriate muscle selection. Since it has a high correlation compared to conventional sEMG devices, it can be used as an alternative to conventional sEMG devices. The newly developed device may be effective to evaluate and monitor the condition of individuals' muscles during repetitive daily activities or exercise, and it has higher accessibility and portability than do conventional sEMG devices.

## Figures and Tables

**Table 1:** Reliability comparison of the Biodex System 3 PRO and BTS-FREEEMG, PSL-EMG-Tr1.

Device	Variable		Trial 1	Trial 2	ICC	Difference of means (95% CI)	*p* value
*(a) Biceps brachii muscle*							
MVIC	Biodex (N·m)	Week 1	36.65 ± 14.56	35.98 ± 13.78	0.985	0.68 (−0.9, 2.3)	0.387
		Week 2	38.60 ± 14.53	38.59 ± 14.27	0.986	0.01 (−1.6, 1.6)	0.990
	BTS-FREEEMG (*μ*V)	Week 1	460.50 ± 257.49	395.52 ± 216.69	0.930	64.98 (14.4, 115.5)	0.014^*∗*^
		Week 2	470.34 ± 243.38	413.91 ± 208.48	0.848	56.43 (−18.7, 131.5)	0.132
	PSL-EMG-Tr1 (*μ*V)	Week 1	398.07 ± 231.36	435.05 ± 281.16	0.937	−36.98 (−96.3, 22.3)	0.207
		Week 2	392.39 ± 227.36	435.65 ± 268.89	0.875	−43.26 (−120.4, 33.9)	0.255
80% MVIC	Biodex (N·m)	Week 1	29.11 ± 11.65	28.52 ± 10.66	0.992	0.60 (−0.3, 1.5)	0.197
		Week 2	31.10 ± 11.18	30.95 ± 11.52	0.986	0.15 (−1.1, 1.4)	0.807
	BTS-FREEEMG (*μ*V)	Week 1	364.27 ± 220.15	333.32±183.99	0.934	30.95 (−15.2, 77.1)	0.177
		Week 2	387.76 ± 220.15	356.38 ± 192.92	0.859	31.08 (−37.13, 99.3)	0.352
	PSL-EMG-Tr1 (*μ*V)	Week 1	304.71 ± 198.40	354.03 ± 218.37	0.872	−49.32 (−115.1, 16.4)	0.132
		Week 2	324.07 ± 185.61	368.97 ± 233.94	0.916	−44.90 (−97.6, 7.8)	0.090
40% MVIC	Biodex (N·m)	Week 1	14.41±5.74	14.55 ± 5.09	0.986	−0.14 (−0.8, 0.5)	0.645
		Week 2	15.48 ± 5.72	15.75 ± 6.00	0.994	−0.28 (−0.7, 0.2)	0.199
	BTS-FREEEMG (*μ*V)	Week 1	118.42 ± 81.22	101.81 ± 75.70	0.920	16.61 (−2.5, 35.7)	0.084
		Week 2	121.36 ± 75.72	115.91 ± 69.50	0.906	5.45 (−14.8, 25.7)	0.579
	PSL-EMG-Tr1 (*μ*V)	Week 1	96.69 ± 67.98	94.32 ± 57.30	0.922	2.36 (−14.2, 19.0)	0.768
		Week 2	94.98 ± 53.06	116.68 ± 72.75	0.832	−21.81 (−43.1, −0.5)	0.045^*∗*^

*(b) Rectus femoris muscle*							
MVIC	Biodex (N·m)	Week 1	147.80 ± 40.50	154.08 ± 36.38	0.948	−6.28 (−13.9, 1.4)	0.103
		Week 2	159.18 ± 46.04	160.05 ± 45.95	0.981	−0.88 (−6.8, 5.1)	0.762
	BTS-FREEEMG (*μ*V)	Week 1	147.82 ± 65.95	144.25 ± 59.11	0.945	3.58 (−10.0, 17.2)	0.588
		Week 2	175.99 ± 59.53	156.78 ± 59.53	0.864	19.21 (−2.7, 41.1)	0.082
	PSL-EMG-Tr1 (*μ*V)	Week 1	123.96±49.66	124.87 ± 55.45	0.899	−0.91 (−16.1, 14.3)	0.901
		Week 2	129.90 ± 52.05	138.30 ± 56.79	0.907	−8.40 (−23.2, 6.4)	0.249
80% MVIC	Biodex (N·m)	Week 1	115.58 ± 31.82	121.67 ± 30.34	0.964	−6.09 (−10.9, −1.3)	0.015^*∗*^
		Week 2	127.06 ± 35.52	126.45 ± 35.75	0.977	0.61 (−4.5, 5.7)	0.805
	BTS-FREEEMG (*μ*V)	Week 1	118.59 ± 80.28	115.13 ± 51.59	0.910	3.47 (−15.0, 21.9)	0.699
		Week 2	136.71 ± 67.52	123.54 ± 48.67	0.898	13.21 (−2.9, 29.3)	0.103
	PSL-EMG-Tr1 (*μ*V)	Week 1	95.46 ± 57.58	101.13 ± 49.94	0.922	−5.66 (−19.3, 8.0)	0.396
		Week 2	100.46 ± 41.52	109.22 ± 45.45	0.943	−8.76 (−17.6, 0.1)	0.051
40% MVIC	Biodex (N·m)	Week 1	57.70 ± 15.90	61.50 ± 15.37	0.956	−3.80 (−6.3, −1.3)	0.005^*∗*^
		Week 2	63.59 ± 18.34	63.81 ± 17.74	0.979	−0.23 (−2.7, 2.2)	0.851
	BTS-FREEEMG (*μ*V)	Week 1	43.45 ± 17.65	44.53 ± 16.57	0.931	−1.08 (−5.2, 3.0)	0.590
		Week 2	50.89 ± 23.43	48.05 ± 21.21	0.883	2.84 (−4.0, 9.6)	0.393
	PSL-EMG-Tr1 (*μ*V)	Week 1	36.15 ± 14.45	39.43 ± 18.16	0.814	−3.28 (−9.3, 2.8)	0.271
		Week 2	38.44 ± 15.68	41.24 ± 18.17	0.957	−2.80 (−5.8, 0.2)	0.070

Values are number or mean ± SD. MVIC, maximum voluntary isometric contraction; ICC, intraclass correlation coefficients; paired *t*-test, ^*∗*^*p* < 0.05; CI, confidence interval.

**Table 2:** Normalization of RMS and reliability of BTS-FREEEMG and PSL-EMG-Tr1.

	Variable		Trial 1	Trial 2	ICC	Difference of means (95% CI)	*p* value
*(a) Biceps brachii muscle*							
80% MVIC (%)	BTS-FREEEMG	Week 1	78.92 ± 13.00	84.84 ± 18.92	0.321	−5.93 (−15.6, 3.7)	0.213
		Week 2	81.44 ± 16.97	84.09 ± 19.76	0.185	−2.65 (−14.2, 8.9)	0.637
	PSL-EMG-Tr1	Week 1	76.10 ± 14.83	82.08 ± 19.99	0.030	−5.98 (−17.8, 5.9)	0.306
		Week 2	83.14 ± 15.51	82.62 ± 17.93	0.246	0.53 (−11.1, 12.2)	0.925
40% MVCI (%)	BTS-FREEEMG	Week 1	27.52 ± 10.17	26.83 ± 10.89	0.862	0.69 (−2.8, 4.2)	0.683
		Week 2	26.86 ± 9.62	28.01 ± 11.22	0.662	−1.14 (−6.1, 3.8)	0.636
	PSL-EMG-Tr1	Week 1	26.07 ± 9.39	24.43 ± 10.10	0.831	1.63 (−1.9, 5.2)	0.350
		Week 2	26.07 ± 9.49	28.16 ± 11.58	0.307	−2.09 (−8.4, 4.3)	0.500

*(b) Rectus femoris muscle*							
80% MVIC (%)	BTS-FREEEMG	Week 1	76.46 ± 18.41	79.91 ± 12.59	0.509	−3.45 (−11.9, 5.0)	0.406
		Week 2	76.70 ± 12.19	78.22 ± 10.13	0.736	−1.53 (−6.4, 3.3)	0.517
	PSL-EMG-Tr1	Week 1	74.87 ± 19.24	80.50 ± 11.95	0.547	−5.63 (−13.9, 2.7)	0.171
		Week 2	77.56 ± 14.27	78.81 ± 10.54	0.580	−1.25 (−7.7, 5.2)	0.688
40% MVCI (%)	BTS-FREEEMG	Week 1	30.43 ± 7.32	32.11 ± 7.78	0.870	−1.68 (−4.0, 0.7)	0.152
		Week 2	30.07 ± 8.18	31.07 ± 8.06	0.752	−1.01 (−4.4, 2.4)	0.545
	PSL-EMG-Tr1	Week 1	29.93 ± 7.93	32.35 ± 8.23	0.765	−2.42 (−5.7, 0.8)	0.132
		Week 2	30.69 ± 7.89	30.84 ± 7.93	0.828	−0.15 (−3.0, 2.7)	0.912

Values are number or mean ± SD. MVIC, maximum voluntary isometric contraction; ICC, intraclass correlation coefficients; paired *t*-test, ^*∗*^*p* < 0.05; CI, confidence interval.

**Table 3:** Technical specifications of newly developed sEMG device (PSL-EMG-Tr1; PhysioLab Co., Ltd., Busan, Korea) and BTS-FREEEMG (BTS-FREEEMG1000; BTS Bioengineering, Milan, Italy).

	PSL-EMG-tr1	BTS-FREEEMG1000
	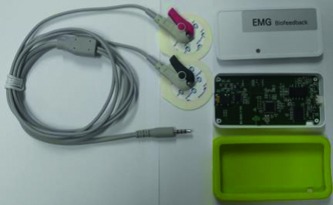	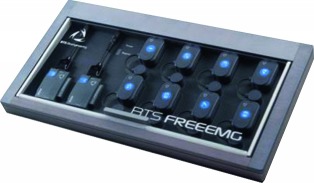

Price	∼$500 USD	∼$25,000 USD

Dimensions (mm)	48 L × 93 W × 15.5 H Lead wire length 1,100 mm (main 500 mm; branch 600 mm)	EMG probes: 41.5 L × 24.8 W × 14 H main electrode Ø 16 × 12 satellite electrode
		USB receiver: 82 L × 44 W × 22.5 H
		Charger: 350 L × 185 W × 20 H

Weight (g)	EMG device: 47 g Lead wire: 41 g	EMG probes: 10 g
		USB receiver: 80 g
		Charger: 1450 g

Channels	1 channel	Up to 10 wireless probes
Bandwidth (Hz)	3–2,000	25–500 Hz
Gain (V/V)	25	2,000
Sampling rate (Hz)	30,000	1,000
Common mode rejection (dB)	73	92

## Data Availability

The data used to support the findings of this study are available from the corresponding author upon request.
